# Serial MRI Imaging Reveals Minimal Impact of Ketogenic Diet on Established Liver Tumor Growth

**DOI:** 10.3390/cancers10090312

**Published:** 2018-09-05

**Authors:** Frances L. Byrne, Stefan R. Hargett, Sujoy Lahiri, R. Jack Roy, Stuart S. Berr, Stephen H. Caldwell, Kyle L. Hoehn

**Affiliations:** 1 School of Biotechnology and Biomolecular Sciences, University of New South Wales, Sydney, NSW 2052, Australia; frances.byrne@unsw.edu.au; 2 Department of Pharmacology, University of Virginia, Charlottesville, VA 22908, USA; sh4hq@virginia.edu (S.R.H.); sujoy.lahiri@gmail.com (S.L.); 3 Department of Radiology, University of Virginia, Health System, Charlottesville, VA 22908, USA; jrr5a@virginia.edu (R.J.R.); SB4b@virginia.edu (S.S.B.); 4 Department of Medicine, University of Virginia, Charlottesville, VA 22908, USA; shc5c@virginia.edu

**Keywords:** liver cancer, ketosis, diethylnitrosamine

## Abstract

Rodent models of liver tumorigenesis have reproducibly shown that dietary sugar intake is a powerful driver of liver tumor initiation and growth. In contrast, dietary sugar restriction with ketogenic diets or calorie restriction generally prevents liver tumor formation. Ketogenic diet is viewed positively as a therapeutic adjuvant; however, most ketogenic diet studies described to date have been performed in prevention mode rather than treatment mode. Therefore, it remains unclear whether a ketogenic diet can be administered in late stages of disease to stall or reverse liver tumor growth. To model the clinically relevant treatment mode, we administered a ketogenic diet to mice after liver tumor initiation and monitored tumor growth by magnetic resonance imaging (MRI). Male C57BL/6 mice were injected with diethylnitrosamine (DEN) at 2 weeks of age and fed a chow diet until 39 weeks of age, when they underwent MRI imaging to detect liver tumors. Mice were then randomised into two groups and fed either a chow diet or switched to a ketogenic diet from 40–48 weeks of age. Serial MRIs were performed at 44 and 48 weeks of age. All mice had tumors at study completion and there were no differences in total tumor burden between diet groups. Although a ketogenic diet has marked protective effects against DEN-induced liver tumourigenesis in this mouse model, these data demonstrate that ketogenic diet cannot stop the progression of established liver tumors.

## 1. Introduction

Primary liver cancer, of which hepatocellular carcinoma (HCC) is the most common form, is the sixth most common cancer, the third leading cause of cancer-related deaths, and is a leading cause of death for patients with cirrhosis [1]. Chronic liver diseases, including hepatitis B and C, aflatoxin B1, and nonalcoholic fatty liver disease, promote cirrhosis and subsequently hepatocellular adenoma (HCA) and HCC development [1]. Metabolic diseases including obesity and diabetes are also important risk factors for HCC, and are likely contributors to the increasing prevalence of HCC in the developed world [1].

Treatment options for liver cancer include transplantation, surgical resection, image-guided ablation, chemoembolization, and systemic therapies including antivirals and the VEGFR/multityrosine-kinase inhibitor sorafenib. Numerous clinical trials have compared the efficacy of similar agents, including lenvatinib and regorafenib, but sorafenib remains the only approved first-line treatment for HCC [2]. Unfortunately, sorafenib only improves survival rates by months [1], highlighting the urgent need for better treatment options for primary liver cancer.

The prospect of utilising dietary interventions to reverse or prevent cancer growth has garnered much interest. One such dietary intervention is the ketogenic diet, comprised of high fat and very low carbohydrate, which leads to the production of ketone bodies (ketogenesis) [3]. The mammalian liver is the main source of ketone bodies, which are produced via fatty acid oxidation-induced production of acetyl-coenzyme A (CoA). When carbohydrate supply to the liver is low, acetyl-CoA is metabolised to acetoacetate (AcAc) and then reduced to β-hydroxybutyrate. A ketogenic diet comprised of medium-chain triglycerides is currently used as a treatment for epilepsy [4]. This diet and the classical ketogenic diet may also prove useful for other disorders including Alzheimer’s disease, diabetes, nonalcoholic fatty liver disease, and cancers of the central nervous system [3,4]. 

The therapeutic potential of the ketogenic diet has been explored for several malignancies in vitro and in vivo [3] and trialed in some patients with advanced disease [5]. However, to our knowledge, no studies have yet investigated whether a ketogenic diet may be beneficial for the treatment of primary liver cancer. Our previous studies have shown that both male [6] and female [7] mice treated with diethylnitrosamine (DEN) at 2 weeks of age were markedly protected from liver tumorigenesis when fed the same ketogenic diet used herein from 6 weeks of age. The aim of the current study was to determine whether ketogenic diet administered after tumor initiation could stall or reverse tumor progression.

## 2. Results

### 2.1. Diethylnitrosamine-Induced Liver Tumourigenesis

In this study, we used the DEN model of liver tumorigenesis in mice. DEN is a procarcinogen that is metabolized by cytochrome P450 enzymes in the liver to produce reactive oxygen species and an active carcinogen that forms mutagenic DNA alkylation adducts [8]. Progressive rounds of cell death and regeneration lead to the development of hepatocellular adenoma that progresses to HCC. A global gene expression comparison study using seven different mouse models of liver cancer found the DEN model to be most similar to HCC tumors from patients with the poorest survival [9]. 

**Figure 1:**
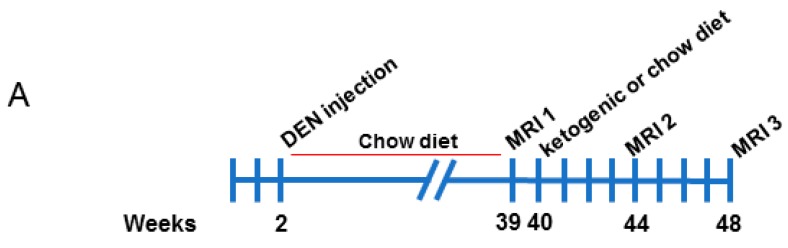

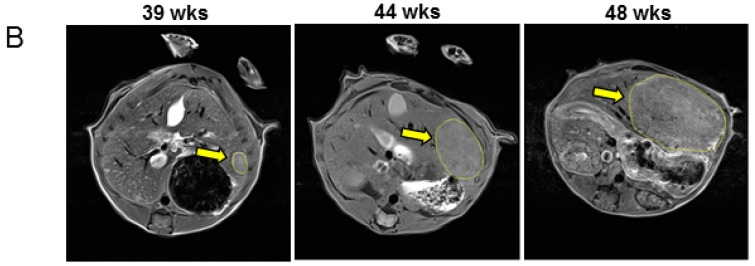
Magnetic resonance imaging analyses of diethylnitrosamine-injected mice fed chow and ketogenic diets. (**A**) Schematic overview of the experimental study. (**B**) Representative serial images of a mouse liver from the chow-fed control group at each MRI timepoint.

Male C57BL/6 mice were injected with 25 mg/kg DEN at 2 weeks of age. Mice were weaned at 3 weeks of age and maintained on a standard chow diet from 3 to 39 weeks of age, then assessed for tumor burden by magnetic resonance imaging (MRI) (illustrated in Figure 1A). Mice were randomised into two groups with similar tumor burden, and half were fed a ketogenic diet while the other half remained on the chow diet. Mice were imaged again at 44 weeks and 48 weeks of age. Representative images of tumors at each MRI scan are shown in Figure 1B. Mice were then culled, and serum and tissues collected.

### 2.2. Validation of Ketogenic Diet Intervention

**Figure 2:**
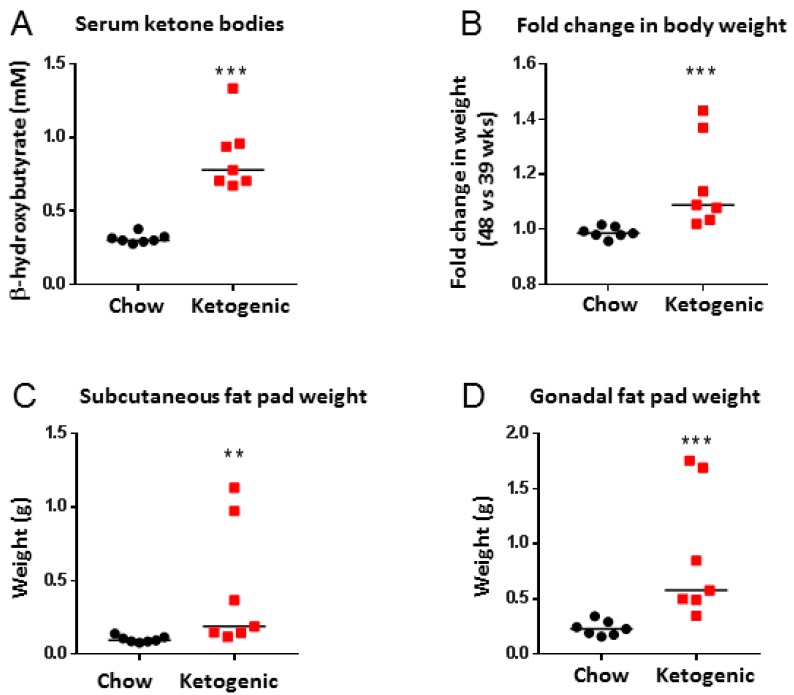
Ketogenic diet-fed mice had increased serum ketones, body weight, and fat weight than chow-fed mice. (**A**) Serum ketone bodies of mice fed a chow or ketogenic diet. Mouse serum was collected at study completion (48 weeks). Data analysed by Mann–Whitney test (*p* < 0.001, bars represent median values). (**B**) Fold change in body weights of mice fed a chow or ketogenic diet (fold change is body weights at 48 weeks divided by body weights at 39 weeks). Data analysed by Mann–Whitney test (*p* < 0.001, bars represent median values). (**C**) Subcutaneous (s.c.) fat pad weights (1 fat pad per mouse) of mice fed chow or ketogenic diet. Data analysed by Mann–Whitney test (*p* = 0.001, bars represent median values). (**D**) Gonadal fat weights of mice fed chow or ketogenic diet. Data analysed by Mann–Whitney test (*p* < 0.001, bars represent median values). *n* = 7 for all analyses.

Sera analyses confirmed that β-hydroxybutyrate levels were significantly higher in mice fed a ketogenic diet compared to chow-fed mice, confirming that the diet induced ketosis (median levels of 0.2997 mM in the chow group vs. 0.7781 mM in the ketogenic group, *p* < 0.001, Figure 2A). Mice fed a ketogenic diet had a significant increase in body weight from week 39 to week 48, compared to chow-fed mice (median fold change chow group = 0.985 vs. median fold change ketogenic group = 1.088, *p* < 0.01, Figure 2B). Mice fed a ketogenic diet also had significant increases in subcutaneous fat (median weight of one fat pad was 0.095 g in chow-fed mice vs. 0.188 g for mice fed a ketogenic diet, *p* < 0.001) and gonadal fat (median fat pad weight of 0.228 g for chow diet vs. 0.578 g for ketogenic diet, *p* < 0.001) compared to chow-fed animals (Figure 2C,D).

### 2.3. Assessment of Tumor Growth

**Figure 3:**
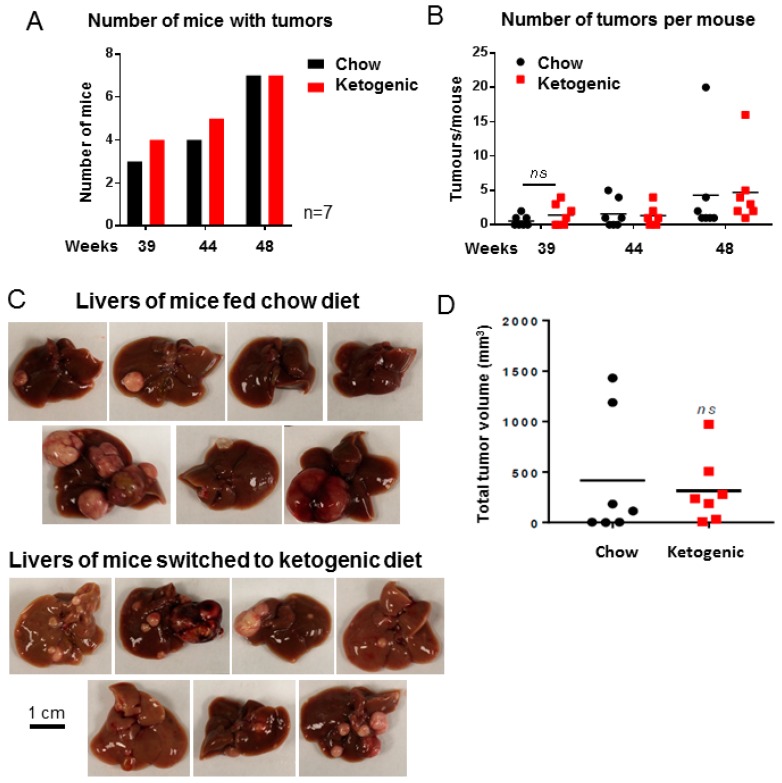
Mice fed a ketogenic diet were not protected from tumor progression. (**A**) Number of mice with tumors visible by MRI at each time point (*n* = 7). (**B**) Number of tumors visible by MRI in each mouse liver (*n* = 7, *ns* = not significant, *p* = 0.9664). (**C**) Images of livers from all mice, fed either a chow or ketogenic diet. (**D**) Total tumor burden per mouse at necropsy (*n* = 7, *ns* = not significant, *p* = 0.7087). Data analysed by two-way ANOVA with Sidak’s multiple comparisons test (alpha = 0.05) for B and unpaired Student’s *t* test for D. Scale bar is 1 cm.

At each MRI time point, both diet groups had similar tumor incidence (number of mice with tumors, Figure 3A,C) and multiplicity (numbers of tumors per mouse, Figure 3B). Images of surface haemorrhaging tumors are shown in Figure 3C for each animal in this study and total tumor burden for each animal is quantified in Figure 3D. Overall, these results demonstrate that a ketogenic diet administered after tumor initiation is not effective at decreasing or preventing DEN-induced liver tumor growth.

## 3. Discussion

Most cancer cells, including liver cancers, undergo metabolic rewiring to increase glycolytic metabolism. This metabolic phenotype was first discovered in rat liver carcinoma tissue by Warburg and colleagues [10]. It is now widely recognized that many types of cancer become dependent on glucose to fuel the high rates of glycolysis. This Warburg-type metabolism is widely regarded as a potential inroad for therapeutic intervention [11]. 

Ketogenic diets restrict glucose availability and limit Warburg-type metabolism. Therefore, ketogenic diets have become increasingly popular in both preclinical animal studies and human studies for their potential to prevent or reverse tumor growth. In general, most clinical and preclinical studies performed to date have shown a beneficial anticancer effect of the ketogenic diet [12,13]. However, some animal models of melanoma, glioblastoma, astrocytoma, and pancreatic cancer were resistant to ketogenic diet, and in some studies, ketogenic diet had a protumor effect in renal cancer and melanoma [12]. In a clinical setting, 42% of studies (10 of 24) showed that a ketogenic diet had antitumor effects, while one study reported a protumor effect and the rest had no effect [12]. Therefore, there are clearly tissue-specific and genetic components that determine whether tumors are sensitive to ketogenic diet therapy.

The DEN model of liver carcinogenesis is the most common model in the field. DEN causes marked genomic instability and produces tumors with heterogeneous mutations and chromosomal gains and losses [14]. This diversity and extent of genotoxic damage is consistent with human HCC with poor prognosis [9]; thus, this model is suitable for investigating whether a ketogenic diet may have anticancer effects that could be translated to the clinic. In our previous studies using the same DEN protocol, a ketogenic diet administered to mice from 6 weeks of age markedly prevented liver tumor formation [6,7]. Therefore, we initially hypothesized that a ketogenic diet administered later during disease progression would have some protective antitumor effects. However, we found that late-onset ketogenic diet administered in the final 8 weeks of the study protocol had no effect on overall tumor burden. This result is consistent with observations by Klement et al., that ketogenic diets are more protective when initiated prior to tumor transplantation [15].

Limitations of this study include the lack of associations between metabolic phenotypes and tumour burden. For example, food intake was not determined as animals were group-housed, and it remains unclear how parameters associated with glucose tolerance, including fed and fasted insulin and glucose levels, or the glucose-ketone index [16], correlate with tumour burden. Furthermore, aging is associated with a decrease in ketone body production [17]; therefore, it is not possible to rule out lesser effects of the ketogenic diet when introduced to mice at 40 weeks of age compared to 6 weeks of age.

## 4. Materials and Methods 

### 4.1. Dietary Intervention in Mice

C57BL/6N male mice were housed and bred in a temperature-controlled room (22 °C) on a 12-h light/dark cycle in filter-top cages and with ad libitum access to food and water. Mice were injected with diethylnitrosamine (DEN) (25 mg/kg) at 2 weeks of age via intraperitoneal (i.p.) injection and weaned at 21 days of age. Mice were maintained on chow diet until 39 weeks of age and then switched to ketogenic diet or kept on chow diet at week 40. Ketogenic diet was prepared in-house and comprised of (w/w) 4% Mineral Mix AIN-76 (Harlan Teklad), 1% Vitamin Mix AIN-76 (Harlan Teklad), 0.4% choline bitartrate, 0.3% methionine, 2% gelatine, 71% lard, 16% casein, and 6% safflower oil as previously described [6], with energy (% by kcal) for protein, fat, and carbohydrate of 8%, 90%, and 2%, respectively. Chow diet was Teklad LM-485 mouse diet, 7012 (Envigo) with energy (% by kcal) for protein, fat, and carbohydrate of 25%, 17%, and 58%, respectively. 

### 4.2. MRI of Mouse Livers

MRI scans were performed at weeks 39, 44, and 48 using a 7T Clinscan MR system (Bruker, Ettlingen, Germany). For radiofrequency (RF) transmission and reception, a birdcage RF coil was used that has a diameter of 35 mm. Heart rate, respiration, and core body temperature were monitored using a fiber optic, MR-compatible system (Small Animal Imaging Inc., Stony Brook, NY, USA). Anaesthesia was maintained using 1.25% isoflurane in O_2_ inhaled through a nose cone. After the mice were anesthetized, gadolinium (Gd) was administered (gadobenate dimeglumine injection, MultiHance, Bracco Diagnostics) in solution at 0.6 mmol/kg via intramuscular injection into the left quadricep. Mice were then held for 5 min for the tracer to circulate. Images were acquired with a T1-weighted, gadolinium-enhanced spin-echo pulse sequence. Imaging parameters were TR (repetition) = 797 ms, TE (echo) = 15 ms, 2 averages, matrix of 192 × 192 (zero-filled to 256 × 256), FatSat (strong mode), 21 slices with 100% gap. This was repeated with 21 adjacent slices to provide a contiguous set of 42 images. Raw DICOM files were sorted and renamed using DICOMSort and then images were imported into ImageJ Fiji to count liver tumors. Minimum detectable tumor size was 1.5 mm in diameter.

### 4.3. Collection of Mouse Tissues and Blood

At 48 weeks of age, mice were euthanized via cervical dislocation and blood collected by cardiac puncture. Blood samples were centrifuged at 5000 rpm for 15 min at 4 °C and serum stored at −80 °C until further analyses. Mouse livers were excised, imaged, weighed, and then surface hemorrhaging tumors counted. Mouse subcutaneous and gonadal fat pads (one pad each from their right side) were weighed and flash-frozen. All animal experiments were approved by the University of Virginia Animal Care and Use Committee (The ethics code is 3942: ‘Metabolic intervention in cancer.’).

### 4.4. Serum Measurements

A β-Hydroxybutyrate (Ketone Body) Colorimetric Assay Kit (Cayman Chemical) was used to measure serum ketone bodies in blood collected from random-fed mice at harvest, as per the manufacturer’s instructions. 

### 4.5. Statistical Analyses

Data with normal distributions were analysed using parametric tests, and data with unequal variances were analysed using nonparametric tests (indicated within figure legends). All statistical analyses were performed in GraphPad Prism.

## 5. Conclusions

Overall, this study demonstrates that a ketogenic diet does not stop or stall the growth of established tumors; however, this study represents a model system in mice and the results must be interpreted in this context where it is not possible to broadly extrapolate the findings to all humans with genetically diverse tumors. However, results clearly show that this common mouse model of liver tumorigenesis is resistant to a ketogenic diet. These data suggest that ketogenic diets may be most effective when used as a prophylactic intervention.
